# Exploring inclusion in UK agricultural robotics development: who, how, and why?

**DOI:** 10.1007/s10460-024-10555-6

**Published:** 2024-02-29

**Authors:** Kirsten Ayris, Anna Jackman, Alice Mauchline, David Christian Rose

**Affiliations:** 1 School of Agriculture, Policy, and Development, University of Reading, Reading, RG6 6UR UK; 2 Department of Geography and Environmental Science, University of Reading, Reading, RG6 6UR UK; 3 Royal Agricultural University, Stroud Rd, Cirencester, GL7 6JS UK

**Keywords:** Agricultural robotics, Emerging technology ethics, Participation, Responsible innovation, Stakeholder inclusion

## Abstract

The global agricultural sector faces a significant number of challenges for a sustainable future, and one of the tools proposed to address these challenges is the use of automation in agriculture. In particular, robotic systems for agricultural tasks are being designed, tested, and increasingly commercialised in many countries. Much touted as an environmentally beneficial technology with the ability to improve data management and reduce the use of chemical inputs while improving yields and addressing labour shortages, agricultural robotics also presents a number of potential ethical challenges – including rural unemployment, the amplification of economic and digital inequalities, and entrenching unsustainable farming practices. As such, development is not uncontroversial, and there have been calls for a responsible approach to their innovation that integrates more substantive inclusion into development processes. This study investigates current approaches to participation and inclusion amongst United Kingdom (UK) agricultural robotics developers. Through semi-structured interviews with key members of the UK agricultural robotics sector, we analyse the stakeholder engagement currently integrated into development processes. We explore who is included, how inclusion is done, and what the inclusion is done for. We reflect on how these findings align with the current literature on stakeholder inclusion in agricultural technology development, and suggest what they could mean for the development of more substantive responsible innovation in agricultural robotics.

## Introduction

The global agricultural sector has been criticised as environmentally unsustainable, as it contributes significantly to greenhouse gas (GHG) emissions (Stern 2006; Pandey and Agrawal 2014), and also to exceeding several of the planetary boundaries (Campbell et al. 2017). In addition, there are economic sustainability challenges for agriculture in many parts of the world, from farm business viability to labour availability, as well as social sustainability challenges such as aging farmer populations (Jöhr 2012; FAO 2020) and mental health crises in the agricultural sector (Shortland et al. 2023).

Increasing automation of the sector has been highlighted as one of the ways in which global agriculture may be able to better meet targets for sustainability, as identified within the UN Sustainable Development Goals (United Nations 2015), and the 2022 State of Food and Agriculture report (FAO 2022). Automation is already present in some parts of the agricultural sector, especially in high-income countries, covering a wide range of technologies, including robotics. In particular, autonomous robotic solutions have been identified by institutions, industry, and parts of the agricultural sector as a key area of technology development (Robs4Crops 2021; DEFRA 2022; FAO 2022).

The benefits and drawbacks of agricultural robotics, along with the associated ethical considerations, have been outlined and interrogated by several authors (Daum 2021; Rose et al. 2021; Ryan et al. 2021; Sparrow and Howard 2021; van der Burg et al. 2022; Ayris and Rose 2023). These issues touch on a wide variety of areas, including environmental impacts, data ownership, labour issues, economic factors, and lifestyle changes. Clear in all of these discussions is a common agreement that the adoption and implementation of agricultural robotics is not uncontroversial. To address the ethical challenges inherent in the development of agricultural robotics, calls have been made for principles of responsible innovation and just transitions to be integrated into agricultural technology development processes (Rose and Chilvers 2018; Eastwood et al. 2019; Burg et al. 2019; Rose et al. 2021; Eastwood et al. 2022; Boon et al. 2022a).

Existing work examining the role of agricultural robotics in the future agricultural sector has predominantly addressed technical questions (Adamides et al. 2017; Huuskonen and Oksanen 2019), or implemented farmer surveys to understand attitudes to adoption (Rübcke von Veltheim, Theuvsen and Heise, 2022; Rose and Bhattacharya 2023). A small number of studies have investigated wider perspectives on agricultural robots (Pfeiffer et al. 2021; Spykman et al. 2021; Spykman et al. 2022; Tamirat et al. 2023). These studies have begun to examine the debates emerging around the use of robotics in agriculture, but many approach these discussions within the context of assumed technology adoption. This approach may contextualise ethical issues as barriers to be overcome, rather than adopting a responsible innovation framework that integrates a requirement to change course where necessary (Owen et al. 2012). Other approaches have begun to explore a wider set of methods to engage farming stakeholders, including the co-design of robotic solutions alongside stakeholders to identify those who are often left behind in such processes (Burch and Legun 2021; Legun and Burch 2021; Ditzler and Driessen 2022). However, these approaches largely do not consider the extent to which inclusive engagement activities have been integrated into the processes of robotics developers, and as such, how far innovation within the sector could be deemed “responsible” or “just”.

In this study, we investigate the current state of the art with regard to inclusion in ongoing agricultural robotics development processes in the UK. We first examine the position of robotics in agriculture as an emerging technology for sustainability in the sector, as well as why substantive and meaningful inclusion is important to achieve responsible innovation and just agricultural transitions. We then present the results of a series of semi-structured interviews conducted with key members of the agricultural robotics sector, which aimed to establish what current approaches to achieving inclusion through the participation of stakeholders look like, and to what extent they align with ideals of participation. These interviews considered three core aspects of the participation processes used by developers: who, how, and why. We then discuss the implications of these findings in the context of facilitating responsible innovation within the agricultural robotics sector, and make suggestions for future work to further explore how more substantive inclusion in the development of agricultural robotics could be facilitated.

## Inclusion and participation in agricultural robotics development

### Robotics in agriculture

There are several types of robotic automation already integrated into agricultural systems across the globe, although many of these technologies are concentrated in high-income countries (FAO 2022). Automated technologies for agriculture that have been widely adopted include robotic milking parlours (Rodenburg 2017) and livestock care robots, such as feeding and barn cleaning robots (Gabriel and Gandorfer 2023). Technologies that have been adopted to a lesser extent include unpersoned aerial systems for precision agriculture (Frankelius et al. 2019; Kim et al. 2019), and weeding robots (FAO 2022; Gil et al. 2023), though these technologies are not widespread and some also face legal restrictions and challenges in certain jurisdictions (Reger et al. 2018; Ayamga et al. 2021).

Autonomous solutions have been identified as part of the next wave of adoption for robotic development for agriculture (DEFRA 2022; FAO 2022), in particular, autonomous robotic solutions for crop care and harvesting. Agricultural robots that can operate autonomously in these areas are currently in development. To understand the degree to which these platforms represent a new technology in agriculture, it is important to assess their functions with respect to their level of autonomy. Within robotics, autonomy usually refers to the degree to which a robot can make decisions and act upon them without human intervention. That is, the robot can sense its environment, plan a goal, and implement actions to achieve that goal without high-level control from a human operator (Beer et al. 2014).

Autonomous robots could have significant benefits for the agricultural sector. Their use has the potential to mitigate labour shortages (Duckett 2018); reduce soil compaction (Lagnelöv et al. 2023); reduce chemical inputs through precision applications (Lu and Young 2020); reduce agriculture’s GHG emissions (Pearson et al. 2022); take over dangerous tasks on farm (Sparrow and Howard 2021); and increase demand for digital skills roles in agriculture (Agri-EPI Centre and Hands Free Farm 2022). However, there are also potential drawbacks to the use of agricultural robotics. Their introduction to the agricultural sector could create unemployment by removing traditional agricultural roles (Martin et al. 2022); reproduce the marginalisation of agricultural workers and displace rural labour (Burch and Legun 2021; Sparrow and Howard 2021); entrench the use of chemical inputs, leading to greater damage to soils and continued environmental pollution (Sparrow and Howard 2021); sever cultural connections to the food system (Sparrow and Howard 2021); deplete the pool of experiential farming knowledge (Legun et al. 2022); and amplify inequalities between those who can afford to implement robotics on farm and those who cannot (Ryan et al. 2021).

### Emerging technologies and responsible innovation

An emerging technology can be defined as one that is fast-growing and novel, with the potential for significant socioeconomic impact (Rotolo et al. 2015). The emergence of autonomous robotics for the agricultural sector could well be argued to fall under this definition, given the many potential impacts that have been identified (Rose et al. 2021; Ryan et al. 2021; Sparrow and Howard 2021; van der Burg et al. 2022), and their recent introduction to the sector.

Emerging technologies have been subjects of ethical debate, with the potential to lead to changes in norms and values that ultimately constitute a societal moral shift (Swierstra 2013). As such, approaches to the ethical assessment of the impacts of emerging technologies have emerged, often rooted in future-facing activities aimed at anticipating the consequences of mass adoption (Swierstra and Rip 2007; Brey 2012, 2017). The importance of anticipating impacts of such technologies is underlined by examples of technologies that have failed to become widely adopted in a particular context in the past, including smart metering technology in the Netherlands rejected over concerns of privacy and security (van den Hoven 2013), and the rejection of genetically modified foods in Europe in the 1990s partially over public ethical concerns around the technology (Lassen 2018). In such cases, the failure to adequately consider the potential consequences of a technology in terms of its social and cultural impact, alongside a failure to integrate public opinion into early discussions of technology development, led to a failure in technology adoption (van den Hoven 2013). More recently, focus has shifted to the role of automation and artificial intelligence technologies, driven in part by the proliferation of large language models, and government institutions are beginning to focus on the responsible and just development of these emerging technologies (Department for Science Innovation & Technology, 2023; Hale 2023; The White House 2023).

Responsible innovation offers one approach by which ethically responsible development of emerging technologies could be facilitated by institutions and developers of technology in partnership with those communities impacted by innovations. Responsible research and innovation (RRI) emerged in the 2010s, first defined by Von Schomberg (2011) as a “mutually responsive” approach to innovation processes that would be ethically acceptable, sustainable, and socially desirable (Von Schomberg 2011). Included within this idea of responsible innovation is an aim to foster innovation of benefit to society, within a normative framework of addressing grand challenges, such as those defined within the UN Sustainable Development Goals (United Nations 2015). It particularly acknowledged the consequences of failing to integrate public opinion earlier in technology development processes, and attempted to recognise the limitations of the prior approaches that led to rejections such as that of genetically modified foods (Owen, Macnaghten and Stilgoe, 2012).

RRI has been adopted as a policy approach in several national contexts in Europe – an RRI toolkit was integrated into the Horizon 2020 research programme in Europe; the UK’s Engineering and Physical Sciences Research Council (EPSRC) has developed the AREA framework for RRI; and the Dutch Research Council (NOW) has a programme of Responsible Innovation that has been ongoing since 2008. Within these contexts, responsible innovation therefore becomes a requirement of the pursuit of research and innovation, and a key tool by which innovators are expected to ethically develop technologies. The field has become a significant area of study for the development of agricultural technology, with recent calls for increased integration of social sciences and frameworks such as responsible innovation into agriculture (Burch et al. 2023), particularly within agricultural technologies such as biotechnologies (Bruce and Bruce 2019; Kjeldaas et al. 2022), smart farming innovations (Bronson 2018, 2019), and agricultural robotics (Rose and Chilvers 2018; Eastwood et al. 2019; Rose et al. 2021).

One of the seminal responsible innovation frameworks in academia was developed by Stilgoe et al. (2013), based on the four pillars of: anticipation; inclusion; reflexivity; and responsiveness (AIRR). Within this framework, anticipation refers to considering potential consequences; inclusion refers to the involvement of the widest possible range of participants or stakeholders; reflexivity refers to establishing an awareness of the context within which an innovation is being developed; and responsiveness refers to a capacity to change course in accordance with revelations from the rest of the responsible innovation approach – including going so far as to not develop an innovation if the pursuit of the framework has indicated that it would not be responsible (Stilgoe, Owen and Macnaghten, 2013). This framework was derived in order to meet the definition of responsible innovation as “taking care of the future through collective stewardship of science and innovation in the present” (Stilgoe, Owen and Macnaghten, 2013, p. 1570). The notion of collective stewardship in itself links this definition and framework for responsible innovation intrinsically to the involvement of plural actors (Frankelius et al. 2019). As such, inclusion could be considered an intrinsic element of the application of the four pillars of responsible innovation, alongside constituting a pillar in itself. Ten Holter (2022) examines the explicit connection between responsible innovation and participatory design, highlighting the common central pillar of inclusivity for both and calling for lessons from participation to be applied for responsible innovation, to avoid institutional capture of its principles.

### Inclusion and participation for just agricultural transitions

The requirement for a just transition to sustainability for global agricultural systems has been highlighted by many scholars (Blattner 2020; Herrero et al. 2020; Boon et al. 2022a), and is supported by bodies like the Food and Agriculture Organisation of the United Nations and the European Institute of Innovation and Technology. Conceptualisations of what constitutes a just transition place the emphasis on substantive inclusion of society. De Boon et al. (2023) propose a three pronged-framework for just agricultural transitions comprising of (1) distributional justice, exploring the costs and benefits of change and how they are distributed across society; (2) procedural justice, the extent to which societal actors are involved and the principles guiding this; and (3) recognitional justice, the involvement of marginalised societal actors and the inclusion of different knowledge types at different scales. A ‘more-than-human’ or multi-species perspective may also be considered as important for substantive inclusion – for example, some research has examined the impacts of agricultural robotics on animal behaviour, welfare, and human-animal interactions (Bear and Holloway 2019). fig. 2

Although inclusion is commonly cited as a beneficial activity to enable just and responsible transitions, scholars have criticised the potential for inclusion to be captured and used to attempt to legitimise non-inclusive practices, thus leading to their delegitimisation (Braun and Busuioc 2020). Studies of inclusion practices within agricultural transitions have found a failure to seek insights from a wide range of societal actors beyond farmers and growers (Bronson 2019), a failure to open up conversations about transition trajectories beyond assumed adoption (McGrath et al. 2023), and difficulties in transparently demonstrating how stakeholder views have influenced development processes (Boon et al. 2022b). Given these insights, it is prudent to explore further how inclusion is being done in the development of agricultural robotics, and as such whether that development can be deemed responsible or just.

## Methods

In this study, we first conducted a narrative review of selected items from the participation literature, which was then used to design a series of semi-structured interviews with developers in the UK agricultural robotics sector. The UK was selected as the national context for this research due to the current stage of agricultural robotics development within its agricultural sector. The UK government has been increasingly promoting agricultural robotics as a focal point of agricultural technology development funding (Dimbleby 2021; UKRI 2022; Wallace 2022), while simultaneously incentivising the adoption of these new technologies (DEFRA 2020). Several small-to-medium sized enterprises (SMEs) in the UK are developing robotic technologies for the UK market, and are increasingly prototyping and testing autonomous technologies.

### Participation narrative literature review

The narrative literature review was conducted to elicit themes to guide the design of questions for the semi-structured interviews, and to frame the analysis of the data elicited from the interview process. A narrative review was selected as the most appropriate method by which to elicit the broad understanding required for this study, given the wide scope of the participation literature. This method offers a means by which key literature can be synthesised to provide an interpretation of the subject, but does not necessitate a systematic review of all literature (Greenhalgh et al. 2018; Rust 2020).

In this study, we have used the concepts of inclusion and participation to examine practices of stakeholder engagement within the development of agricultural robotics. We consider inclusion as a goal of responsible innovation aiming to facilitate legitimate and just transitions for agriculture, wherein participation is a mechanism by which this inclusion is achieved (Jones 2011). We focus on participation not as a distinct approach to our research (i.e. we do not use participatory approaches to shape our methodology for this study), but rather as the subject of investigation in itself. We examine the core values of participation (both within and without the agricultural context), as a means by which inclusion can be achieved, and as a process that can open up pathways to avoid the pitfalls of non-inclusive practices.

**Fig. 1:**
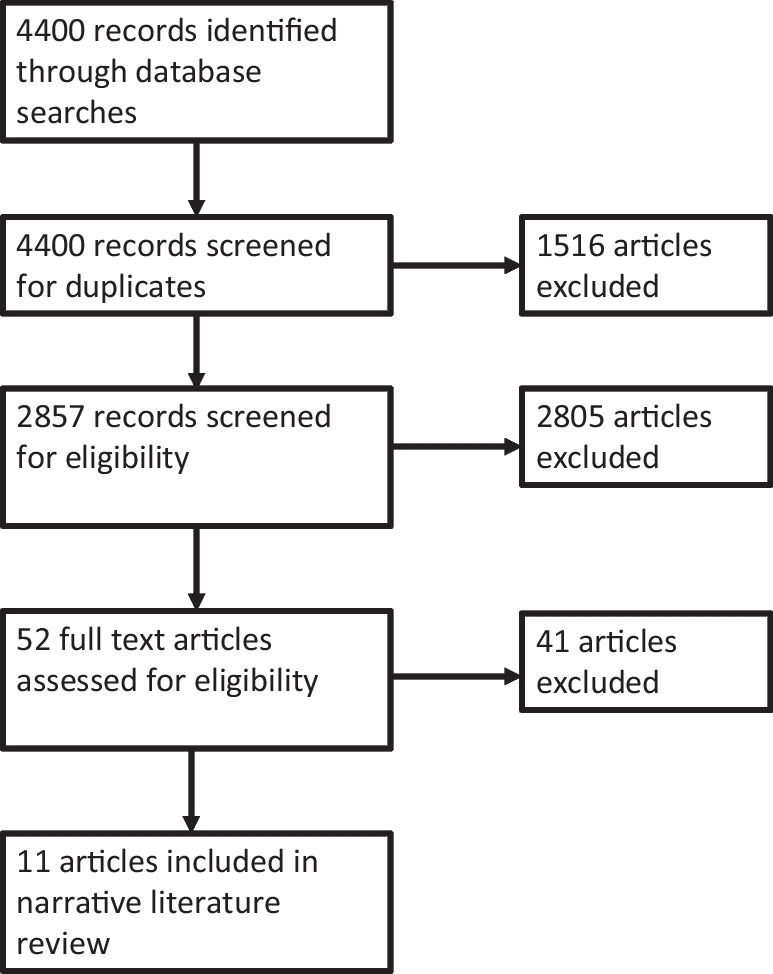
A flow diagram detailing the narrative literature review screening and exclusion process, adapted from the PRISMA flow diagram (Page et al. 2021)

As the aim of this study was to elicit unbiased descriptions of the current processes and methods by which robotics developers in the UK are involving external stakeholders in their technology development, the search terms for the narrative literature review were chosen to reflect the notion of participation as the process by which inclusion is achieved. In order to gather relevant publications for this review, searches were conducted on two key databases: Web of Science and SCOPUS. These searches were defined by selecting key words from a broader review of the participation literature. The search terms used were: participation; public participation; participation AND agriculture; participation AND public engagement; participation AND science and technology studies; participation AND typology; public engagement; stakeholder AND participation; participatory AND decision-making; participatory AND practices; and participatory research. The first 100 results were downloaded from each search per database. These results then went through an initial sifting process, in which results that were not relevant to the search topic were excluded (e.g. medical studies). This sifting process was based on reviewing the titles and abstracts of the papers. The resulting 52 articles were assessed in full to check for eligibility, based on their relevance to the search criteria and their prevalence in the field based on citation count, with adjustments made to account for more recent releases. 11 articles were ultimately included in the narrative literature review – see Fig. 1 for a full accounting of the exclusion process.

This literature review was not conducted as a systematic review (Page et al. 2021), but rather as a systematically approached narrative literature review, identifying a small number of key pieces of participation literature from which to ground the subsequent interviews. As such, the search strategy and screening of results for the review has some limitations, and could be expanded upon to provide a systematically-derived understanding of participation. However, for the purposes of this study, the narrative literature review provided satisfactory context and key themes to deepen the authors’ understanding of the field and situate the remaining work in this study (Greenhalgh et al. 2018). The key learnings from this narrative literature review are presented below, as they informed the design of the semi-structured interviews that are the focus of the results and discussion.

### Distilling the ideal of participation

Four key themes of participation were identified: context; who is included; how is inclusion done (engagement approaches that could be further subdivided into methods and good practice); and what is inclusion for.

#### Context

The context of participation included characteristics of broad contextual factors, relational factors, temporal factors, framing, and ecologies/networks. These characteristics were highlighted by authors throughout the review as heavily influencing the success of participatory approaches, for example in Bell and Reed’s ‘Tree of Participation’ (2021). Requirements to address contextual factors such as time constraints (temporal factors), or facilitator skills (relational factors) form a central part of Neef and Neubert’s (2011) framework for stakeholder participation in agriculture. Such considerations highlight the importance of understanding the wider social and cultural backdrop against which participatory exercises take place. These considerations also align with the reflexivity of the AIRR responsible innovation framework, which aims to introduce context and framing to research and innovation processes.

#### Who is included?

This aspect of participation included characteristics of broad inclusion, scale, and representativeness. The reviewed papers were unanimous in identifying inclusion as a key characteristic of participatory approaches. Unsurprisingly, inclusion featured prominently throughout the participation literature. However, although there was general agreement that attention should be paid to achieving diverse and representative inclusion, the papers did not necessarily agree on how participants could be substantively identified.

Some papers offered insight into methods for facilitating inclusion at only a high level, such as the “controlled” or “uncontrolled” selection mechanisms discussed by Rowe and Frewer (2005), and Neef and Neubert’s brief discussion of methods for identifying a range of stakeholders in participatory agricultural research (Neef and Neubert 2011). Other papers offered a deeper examination of approaches to inclusion, such as Reed’s exploration of a systematic approach for representing relevant stakeholders (Reed 2008), and Chilvers and Kearnes’ call to move beyond non-substantive inclusion into an approach of “ecologizing participation” (Chilvers and Kearnes 2020, p. 358).

#### How is inclusion done?

The engagement approaches that constitute participation were subdivided into methods and aspects of good practice. Characteristics that reflected good practice within participatory approaches had the most variability, with no single characteristic discussed by all of the reviewed papers. However, themes of democracy and equality were common, reflecting the importance of substantive inclusion in participatory approaches (Chilvers and Kearnes 2020; Bell and Reed 2021). Accountability, transparency, and trust were also commonly cited (Reed 2008; Hurlbert and Gupta 2015; Bell and Reed 2021), highlighting the consideration of power dynamics inherent in the participatory literature – in acknowledging the need for clarity and fairness, these papers highlight the potential for participation to be insubstantial or tokenistic if these practices are not adhered to. Methods for participatory approaches were equally variable, with some placing greater emphasis on dialogue, others on co-production, and others still offering a range of tools that could be used to facilitate participation (Rowe and Frewer 2005).

#### Why is inclusion done?

The purpose of the participation included learning, consensus building, legitimacy, and empowerment. Of these categories, empowerment was the most frequently cited of the purposes of participatory approaches. This reflected the central position of power in the original conception of participation (Arnstein 1969), and remained a strong focus throughout, emphasising the importance of the inclusion of the widest possible range of voices. Learning was also commonly cited as one of the key purposes of participation, most notably in Pretty (1995) and Hurlbert and Gupta (2015), wherein learning is the primary aim of the participatory activity. Consensus building (Innes and Booher 2004; Rowe and Frewer 2005; Hurlbert and Gupta 2015) and the granting of legitimacy to decision-making processes (Rowe and Frewer 2005) were also identified to a lesser extent in the literature.

### Semi-structured interviews – participants and methods

**Fig. 2:**
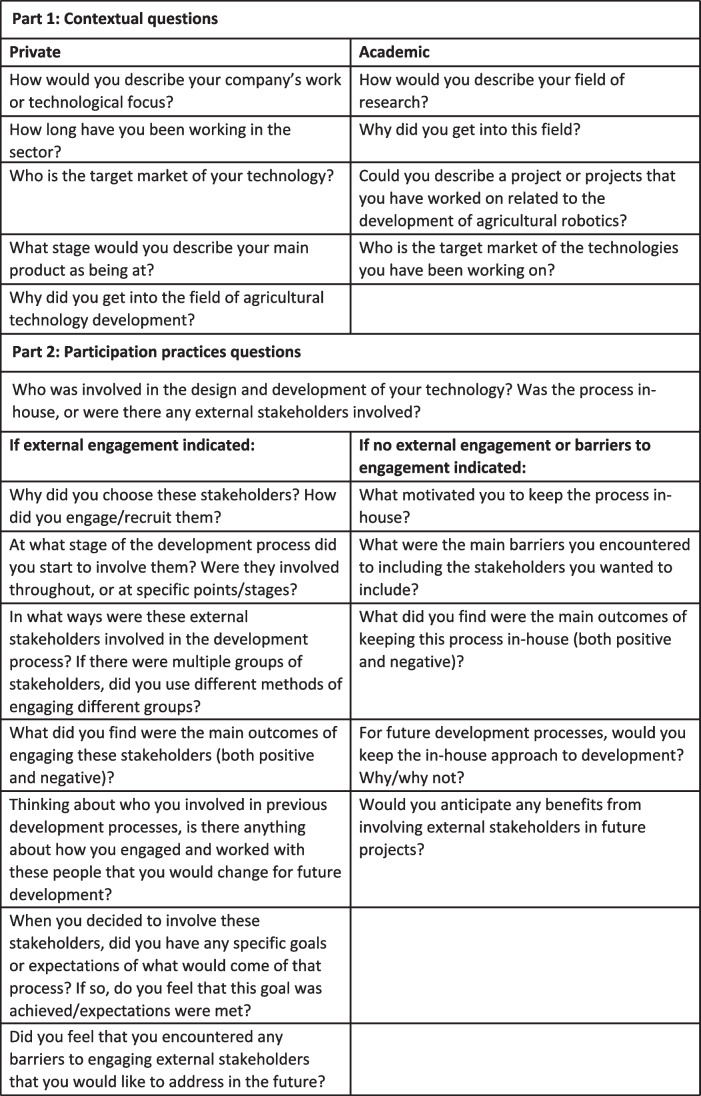
The interview guide used during the semi-structured interviews, divided into ‘Part 1: Contextual questions’ and ‘Part 2: Participation practices questions’

The semi-structured interviews were designed in order to understand the approaches to stakeholder engagement that have been taken by developers currently operating in the UK agricultural robotics sector. The interview guide was devised based on the findings of the narrative literature review of the participation literature (presented in the above Distilling the ideal of participation section), using the four main category themes of context, who is included, how is inclusion done, and what is inclusion for. Questions were divided into those suitable for private developers, and those suited for academics working in agricultural robotics development research, and was further designed with the intention of avoiding any perception of judgment of the engagement approaches described by interviewees. Each interview was divided into questions relating to preliminary background; current engagement practices focusing on the who, how, and why of participation; and reflection. During interviews the questions were used as a guide and, where appropriate, the exact phrasing of the questions was adapted, or particular points were followed up in order to elicit a full picture from the interviewee. Details of the interview guide are provided in Fig. 2.

This study focused specifically on developers working in the UK context. As such, developers working primarily in other national contexts, or members of the UK sector selling a platform in a different national context as a third-party vendor, were excluded. The study also focused on systems developed for field or polytunnel crops – as such, developers working on robotics targeted primarily at livestock were excluded (although some of the participants touched on work with livestock robotics where this was part of their overall experience of agricultural robotics). Both private industrial developers and academic researchers were invited to participate in this study. This approach was selected to reflect the nature of the agricultural robotics sector in the UK. As an emerging field, there are significant connections between private developers and academia. Many developers work alongside academic institutions, and available funding opportunities have encouraged industry collaboration with academia (UKRI 2022). As a result of this close connection between academia and industry in the sector, only a limited distinction was made in the interview guide between academic and industrial participants – in particular, the background questions were adapted but the questions regarding participation practices remained the same for all developers.

Through consultation with two key agricultural technology networks in the UK – the Agri-EPI Centre and Agri-TechE – it was established that the industrial participant list accurately reflected their understanding of the agricultural robotics sector in the UK. Academic participants were identified through institutional membership of the UK-RAS Strategic Task group in Agricultural Robotics. A further snowball sampling approach (Parker et al. 2019) identified 5 additional potential participants not otherwise identified through the aforementioned networks.

A total population size of 24 individuals was identified. These individuals were sent an email invitation to participate in the study, including a participant information sheet. A follow-up email was sent if no response was received. Of the individuals invited to participate, 11 agreed to be interviewed, representing a 45% participation rate. Although the sample size of 11 is small, this represents a significant proportion of the agricultural robotics development community active in the UK at the time of the study, as confirmed by both the initial identification via agricultural technology networks, and the snowball sampling approach taken with participants to identify any members beyond the formal networks.

Interviews were conducted online via Microsoft Teams, Zoom, and Google Meet, recorded, and then transcribed. The transcribed interviews were redacted to remove clear identifying information, and uploaded to NVivo for analysis. Due to the sensitive commercial and personal details revealed through the interview process, the transcripts have not been made available and are instead discussed through key statistics and anonymised quotes. A qualitative analysis of the data was subsequently conducted using a thematic coding approach (Braun and Clarke 2006), and the results are presented in the following section.

## An analysis of participation in UK agricultural robotics development

In the following section, we discuss the results from the semi-structured interviews. As highlighted in the interview guide in Fig. 2, the interviews were divided into two parts. Part 1 was focused on establishing contextual information, while part 2 explored stakeholder participation and inclusion in particular. We focus primarily on analysing the results of part 2 of the interview guide, covering who is included, how inclusion is done, and why inclusion is done. Future work could consider exploring the contextual influences on agricultural robotics development processes more widely, but investigating this was deemed beyond the scope of these interviews due to the narrow contextual view that could be gathered directly from interviewing only roboticists.

### Who is included?

In response to the questions in part 2 of the interview guide (see Fig. 2), each of the interviewees discussed the active inclusion of stakeholders in their work developing robotic systems for agriculture, identifying a range of stakeholders that had been involved in one or more of their development processes. Table 1 details the full range of stakeholder groups that were engaged by interviewees for agricultural robotics development, and the number of individual interviewees who mentioned inclusion of each type of stakeholder group. Inclusion of farmers or growers was discussed by all interviewees, demonstrating a clear trend within the sector for inclusion of these stakeholders.

**Table 1:** Summary of stakeholder groups identified by interviewees as previously or currently engaged in their agricultural robotics development processes

Stakeholder group engaged	Number of interviewees discussed (n = 11)
Farmers/growers	11
Large agricultural companies	5
Technology companies	5
Academics	4
Agronomists	3
Insurers	3
Policymakers	3
Research institutions	3
Supply chain actors	3
Farm managers (e.g. operational, technical)	2
Research governing bodies	2
Agricultural workers (i.e. pickers)	1
Financiers	1
Standards institutions	1

The Ethical Matrix devised by the Food Ethics Council in the UK proposes four groups that should be considered in ethical decision making regarding food issues, and in particular in decisions regarding new technologies for the food and farming sector: people in the food industry; citizens; farm animals; and the living environment (Mepham 2000). Of the stakeholder groups identified by interviewees in this study, all would primarily fall under the first of these categories – people in the food industry. The stakeholders engaged all held a direct stake in agricultural systems – as professionals working in agricultural environments, policymakers and institutional actors with direct influence in agricultural policy, or as members of the supply chain linking agricultural systems to the wider food system (e.g. supermarkets). It is worth noting that each group of stakeholders identified in Table 1 is not homogenous – farmers and growers were included by all interviewees, but a limitation of our analysis is that we do not know their individual characteristics. That is, we do not know whether these were large or small farmers, organic or conventional, etc., but research shows that there can be a tendency to not involve so-called ‘harder-to-reach’ farmers in innovation (Hurley et al. 2022).

**Fig. 3:**
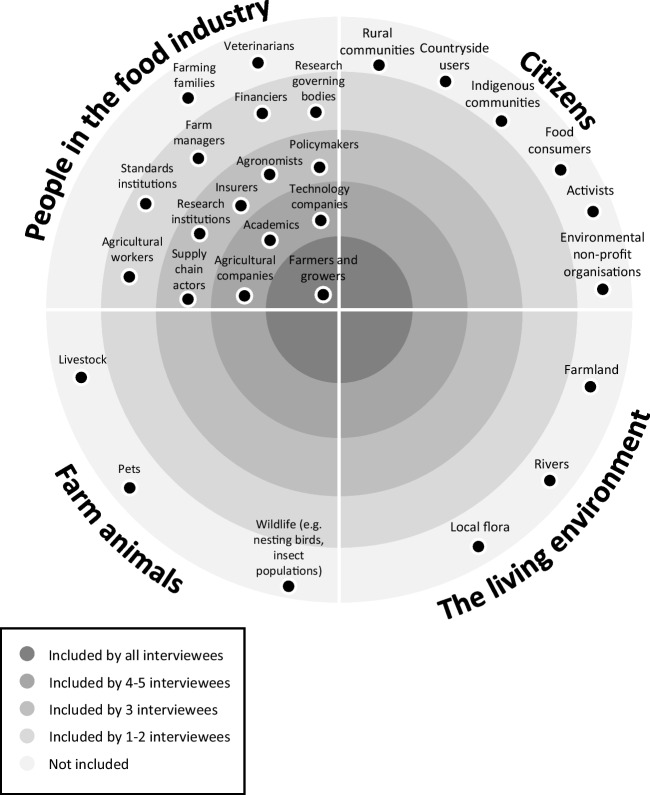
A stakeholder diagram showing the stakeholder groups from most to least included in UK agricultural robotics development, based on the number of times each group was mentioned by interviewees (n = 11). Where examples have been added of stakeholder groups that were not included, these examples are representative groups, and do not constitute an exhaustive list. Based on the Ethical Food Matrix (Mepham 2000)

None of the interviewees highlighted engagement of actors with a more indirect stake in agricultural systems, such as rural communities, food consumers, and local flora and fauna. Some of the identified stakeholders may represent the views of other groups within the matrix (for example, policymakers may represent the living environment), but without further exploration of the exact nature of the engagement processes, such indirect representation cannot be assumed. Figure 3 maps the stakeholders mentioned by interviewees to Mepham’s Ethical Matrix, and highlights examples of stakeholders who were not mentioned. This figure shows that while a large number of stakeholders have been involved in agricultural robotics development processes in the UK, there is a lack of diverse representation from the categories of citizens, farm animals, and the living environment. Attention should also be paid to stakeholders who have not been included in the category of people in the food industry – strong overall representation in this category does not necessarily constitute saturation of the stakeholders who could, and should, be consulted.

A further point to highlight is the paucity of more-than-human actors (i.e. farm animals and the living environment) within Fig. 3. While it is true to say that these stakeholders were not mentioned by interviewees during this study, it is important to note that the non-directive approach of the interviews avoided specific prompting about which stakeholders were included. As such, interviewees may have not mentioned engagement of these more-than-human actors due to an interpretation of the interview questions as focusing exclusively on human stakeholders. It is therefore not possible to categorically report from these data that more-than-human actors were excluded from development processes. However, as none of the interviewees mentioned more-than-human actors when asked about stakeholders, this demonstrates that interviewees did not associate more-than-human actors with stakeholder engagement, so it remains likely that these stakeholder groups remain underrepresented.

Participants were less able to identify stakeholders that they would be keen to include in future development processes. Table 2 shows a smaller range of potential targets for future stakeholder engagement, with most interviewees able to identify only one or two stakeholder groups for future inclusion, and three interviewees who did not identify any other stakeholders for future involvement.

**Table 2:** Summary of stakeholder groups identified by interviewees as potential targets for future stakeholder engagement in their agricultural robotics development processes

Stakeholder group for future engagement	Number of interviewees discussed
None suggested	3
Agricultural workers	2
Supermarkets	2
Technology companies	2
Downstream customers	1
Health and Safety Executive	1
Policymakers	1
Standards institutions	1

The stakeholder groups identified as desirable groups for future engagement were often a step removed from pre-farm gate production, with the exception of agricultural workers. Interviewees identified stakeholders such as policymakers, supermarkets, and the Health and Safety Executive (HSE). These stakeholders share a key characteristic of having high influence in the wider food system within which the developers are aiming to introduce their technologies. The only lower influence stakeholders identified were agricultural workers and downstream customers.

Innovation in agricultural technology research and development has been criticised for a narrow approach to the ‘who’ of inclusion (Rose and Chilvers 2018). The results of this study corroborate this criticism, demonstrating that inclusion in agricultural robotics development is predominantly restricted to the end user, defined largely as the farmer/grower. It is important to note that the notion of the “end user” in these engagements often referred to the farmer or manager that would be purchasing the technology, and not necessarily to a worker (such as a labourer, or agronomist) who might use the robot. In some cases, this may be because the robotic technology is intended to replace the worker, and thus the “end user” is the person managing the field. However, there are other cases in which agricultural workers or external agricultural consultants may be working with or alongside the robotic technology, thus highlighting the need for engagement of a wider range of “end users” than is seen in this study.

Where other stakeholders are involved, these stakeholders are typically high influence (e.g. policymakers with power over legislative issues), or are involved in the process as a development collaborator (e.g. research institutions or other technology developers). In a “power vs interest” stakeholder mapping grid, the stakeholders engaged by the interviewees were either “subjects” (high interest, low influence) or “players” (high interest, high influence), leaving those with low influence without a voice in robotics development processes (Bryson et al. 2002; Ahmadi et al. 2019). These low influence stakeholders largely fall into the three categories of Mepham’s Ethical Matrix in Fig. 3 that are currently often being excluded from development processes.

Interviewees were also asked about barriers to inclusion that they had encountered during their development processes. The most frequently identified barriers are summarised in Table 3. The barriers identified by interviewees are predominantly focused on stakeholders on-farm – i.e. farmers and growers, and agricultural labourers. When discussing barriers encountered with farmers and growers, interviewees spoke of the time pressures of farm management, aligning with previous findings that highlight time constraints as one of the key barriers to engagement (Jansen et al. 2010; Hurley et al. 2022; Schillings et al. 2023).

**Table 3:** Summary of the most frequently cited codes identified within the ‘barriers’ theme, including sample quotations that illustrate the code

Barrier	Number of interviewees discussed	Sample quotations
Language barrier	4	***“****[we are now doing] a semi-structured interview with the translator in place because that’s what often is needed, there’s a communication barrier sometimes” ****ARI114***
Lack of interest	3	***“****in the end to really get [the stakeholders] to see the value, when you talk about several relatively low TRLs [Technology Readiness Levels] they must have a certain passion to be actually be actively engaged” ****ARI114***
Time pressures	3	***“****But then contacting [the farmers] again after the show – nothing. It’s kind of like that was their free time, in a way, they’re happy to chat, but once they go back to the farm, you know, especially in season…” ****ARI123***

Barriers were also identified to engaging agricultural workers in development processes. One interviewee spoke about including agricultural workers via representative organisations, while others discussed the language barriers encountered when attempting to engage with agricultural workers. Similar issues have been identified with language barriers previously in agriculture for the safety and dignity of agricultural workers (Consterdine and Samuk 2015, 2018), as well as in other societal contexts for participatory engagement (Watkins et al. 2012; Schinkel et al. 2019). The limited involvement of agricultural workers also reflects the findings of Burch and Legun (2021), who further discuss the relational barriers to substantive engagement of agricultural workers that were not mentioned by interviewees in this study. As such, should the language barrier be addressed, there would still be significant work to be done in establishing relationships and providing the time and space for agricultural workers to engage meaningfully in the development of agricultural robotics.

As demonstrated in Fig. 3, it is not only harder-to-reach farmers and growers (Hurley et al. 2022), nor agricultural workers, who are not being included in agricultural robotics development processes in the UK. There is a wider absence of participants from the “citizen” group of Mepham’s Ethical Matrix – non-agricultural actors who nonetheless are an intrinsic part of the food system as consumers of food. The potential reasons for the exclusion of these stakeholders are discussed further later in this section.

### How is inclusion done?

Interviewees were invited to speak about the nature of the engagement activities they had used during their development processes, and reflect both on the tangible methods they used and the qualities that they considered to be important in those methods.

Interviewees mainly identified networking as their initial approach method for including farmers and growers in their development – both formal and informal. The predominance of this approach may offer some explanation of the narrow scope of inclusion identified in this study. Table 4 summarises the methods interviewees used to approach stakeholders for engagement. Given the relatively small number of farmers engaged by each developer, it is likely that by using networks as methods by which to achieve engagement, harder-to-reach farmers are being excluded from participation in development of these technologies (Hurley et al. 2022). Involving these harder-to-reach stakeholders is crucial to address uneven engagement in agricultural technology development (Bronson 2019) and broaden the sector’s approach to inclusion in line with the principles of responsible innovation (Rose and Chilvers 2018).

**Table 4:** Summary of the most frequently cited codes identified within the ‘inclusion approaches’ theme, including sample quotations that illustrate the code

Category	Number of interviewees discussed	Sample quotations
Networks (formal)	7	*“KTN [Knowledge Transfer Network] networking events were really good also, the sort of kick-off presentations that you get from the TSB [Technology Strategy Board] or Innovate UK was also very useful, especially in the early days because nobody had had done it at all at that point” ****ARI122***
Cold-calling	3	*“…then we basically just reached out to growers cold. Well, it wasn’t pure cold calling, but not far off, you know, just reaching out” ****ARI123***
Networks (informal)	3	*“And then just kind of one thing leads to the other, someone has a connection for you for a poultry farm, and then you get to speak to some apple farmers” ****ARI102*** *“those people [who don’t turn up to events] are people you end up engaging with more on a personal level i.e. when I’m down the pub and you’re speaking to someone about what you do” ****ARI117***

Once stakeholders had been contacted and confirmed their interest in participating, interviewees spoke about the methods they used to facilitate engagement, both in terms of practical methods, and the good practice values that informed their implementation. Table 5 summarises the most frequently cited good practice values that interviewees attributed to their engagement methods. Most discussed was the early involvement of stakeholders in the development process. The timing of involvement is a key consideration within responsible innovation, where the Collingridge dilemma – wherein early involvement may come before a technology is developed enough for consequences to be imaginable, but late involvement may come too late for meaningful change (Collingridge 1980) – demonstrates the criticality of timing the participation of stakeholders well.

**Table 5:** Summary of the most frequently cited codes identified within the ‘good practice’ theme, including sample quotations that illustrate the code

Good practice (based on themes from the literature review)	Number of interviewees discussed	Sample quotations
Early involvement	6	*“…even before you start your project, you figure out what is it [the customers] actually want” ****ARI119***
Transparency	5	*“once [the growers] see their feedback incorporated it kind of builds that trust and builds that – you know that actually this is for my benefit” ****ARI123***
Diverse representation	4	*“everyone’s different, and unless you’re down there seeing it then you kind of don’t understand what [the growers are] doing really” ****ARI123***
Site-specific engagement	4	*“not only do [the farmers] have to see it, ideally they have to see it on their own land” ****ARI107***

One of the key aspects of good practice identified by interviewees was transparency, aligning with the strong trend in the participation literature for transparency, trust, and accountability as a key feature of participatory approaches (Innes and Booher 2004; Reed et al. 2009; Hurlbert and Gupta 2015; Bell and Reed 2021). However, other aspects of good practice highlighted by the narrative literature review are missing from the responses given by interviewees – in particular the emphasis on democracy and equality.

The methods of engagement for most developers constitute interviews and stakeholder meetings, and on-site visits, demonstrations, and trials. These are summarised in Table 6. The methods identified reflect more traditional approaches to stakeholder engagement, but do not necessarily integrate more creative techniques that could be used to elicit more substantive engagement, such as methods explored by Ditzler and Driessen for the design of agroecological farming robots (Ditzler and Driessen 2022). Ditzler and Driessen explore the design of a robot for an agroecological pixel cropping approach to farming through a series of interactive workshops, including a World Café (Brown and Isaacs 2005) with a variety of participant groups, and a design challenge targeting second-year design students (Ditzler and Driessen 2022). There are a variety of other methods of participation, including ethnographic studies; visual methodologies (e.g. film, artwork, poetry, stories), including for future-gazing (Daum 2021; Science and Society Collective, 2023); deliberative workshops (Chilvers and Kearnes 2020); citizen juries or public dialogues (Food Farming and Countryside Commission 2023); and interviews and surveys translated into multiple languages, which can help overcome barriers relating to language and include different knowledge types (Mattila 2021; Bogoeski 2022). In addition, working with local trusted farmers, advisers, and technology networking organisations, as well as engaging in non-traditional settings (pubs, agricultural shows, marts) can make it easier for ‘harder-to-reach' farmers and rural communities to participate (Hurley et al. 2022). Animal welfare scientists and ecologists could help to design studies that capture how animals (livestock and wildlife) interact with robots and how they behave towards them, as a method of facilitating the inclusion of more-than-human participants.

**Table 6:** Summary of the most frequently cited codes identified within the ‘methods’ theme, including sample quotations that illustrate the code

Methods	Number of interviewees discussed	Sample quotations
Technology demonstration	6	***“****Some of the bigger things have actually come from demonstrations of the outputs” ****ARI107***
Farm visits	5	***“****So we’ve been out on harvesters, we’ve been out in the fields and I think that’s a really, really important part of learning the process because it’s so easy to get the wrong end of the stick” ****ARI122***
Stakeholder meetings	5	***“****establish a regular meeting forum with [the farmers] to say well this is where we’ve got to, what do you think” ****ARI113***
Interviews	5	***“****it was a succession of speaking to farmers, speaking to farmers, speaking to farmers” ****ARI102***
Formal events	4	***“****You mention you’re trying to find an answer to the problem […] and you immediately gather a crowd around you, and that happens wherever we go. We’ve been to several shows and demonstrated that” ****ARI113***
User trials	3	***“****then in the final year we actually took our technology and gave it to [the farmer] to use, and then we had a farming partner who then used that technology for a couple of days – well for a week or so on their farm” ****ARI117***

In most cases, interviewees spoke of the inclusion of a specific farmer or grower as a partner in the development of the technology, although one interviewee also spoke of having a board of farming partners advising their development. The involvement of this board reflects a stronger trend of participation, representing participation on a higher rung (partnership) of Arnstein’s traditional ladder of participation (Arnstein 1969). However, in the majority of cases, the traditional approaches to engagement mentioned sit lower on this ladder in an area described by Arnstein as “tokenism”. This may be a result of the limitations of more traditional methods of engagement, wherein participation is motivated by technical questions or problem definition.

Although this study focused on the practices of robotics developers for participation in agricultural robotics, it is important to also consider this subject from the perspective of the participants. Legun and Burch (2021) highlight the criticality of the self-perceived agency of participants. Although the methods highlighted by interviewees offer the potential for substantive participation (particularly through farm visits, technology demonstration, and user trials), these methods also have the potential to focus too closely on solving technical problems, rather than offering participants an active role in developing the technology. Guthman and Butler (2023) have observed similar trends at Silicon Valley-adjacent technology events targeting innovators aiming to expand into the agricultural sector, wherein technologies are presented as solutions before problems are explicitly identified, and developers may fail to engage more holistically with the systematic change required for a just transition to sustainable agriculture. It is therefore crucial to assess how these methods are used, the context within which participants are invited to take part, and the participants own perceptions of their agency or passivity within the development process as a whole.

### Why is inclusion done?

Interviewees were asked about the motivation for the stakeholder engagement that they identified, and Table 7 summarises the most frequently identified reasons for engaging stakeholders in the development of agricultural robotics.

**Table 7:** Summary of the most frequently cited codes identified within the ‘purpose’ theme, including sample quotations that illustrate the code

Category	Number of interviewees discussed	Sample quotations
End-user confidence	7	*“the part around the farmers is—the design part, the engagement with the farmers—is really around the confidence in the machinery” ****ARI107***
Technical requirements	7	*“And now, how do you want this crop to be harvested? Do you want to cut like this or cut like that? Can we break it off?[…] That gives you some very clear guardrails as to what it is that you need to achieve” ****ARI102***
Problem comprehension	7	*“we’ve got to produce something that we think the farming community needs” ****ARI113***
Developer education	5	*“it’s really important to have […] the farmers especially when it comes to developing robotics for farming specifically because I personally think engineers are useless when it comes to farming and likewise, you know, farmers aren’t exactly roboticists” ****ARI120***

The reasons given for pursuing stakeholder engagement align with some of the motives identified in the participation literature – in particular, legitimacy. Several interviewees spoke about the importance of end-user confidence and trust in their product, and highlighted this as one of the outcomes of their engagement with farmers and growers.

Interviewees also discussed the need for engagement in order to understand technical requirements, or to understand the problems that farmers face that could be solved using a robotic system. One interviewee explained the motivation for pursuing this approach:

“that group [of farmers] when we produced the first robot and we ran it in front of them, so the first big robot, […] they fed back pretty much straight away and said […] this looks ridiculous” ARI107

The purpose of stakeholder engagement in these interviews is strongly associated with addressing technical questions – understanding what problems farmers are facing, educating developers to be able to understand the environment in which the technology will be applied, and subsequently instilling confidence in the end-user that the technology is functional and reliable. Mostly, therefore, engagement was aimed at improving technical design to help with adoption, which is the focus of most research studies that claim to have done stakeholder engagement in the area of agricultural technology (McGrath et al. 2023). These identified purposes are similar to those discussed by Eastwood et al. (2019) in their exploration of New Zealand smart dairy farming technologies, wherein they suggest a technocentric approach to innovations in the sector precluded consideration of socio-ethical challenges. This is echoed in what Reisman (2021) describes as the sanitisation of agri-food technology, in which a narrative is built around technologies to emphasise their urgent application in addressing grand challenges. In doing so, time is not devoted to ethical debate regarding development and adoption.

The ethical motivations for pursuing stakeholder engagement are absent in the responses offered by interviewees in this study. In part, this may be due to a perception that the ethical requirement for these technologies, whether for the purpose of addressing labour shortages or mitigating climate change, has already been agreed upon. Such a perception may be due to the narratives that exist within the UK context regarding the use of agricultural robotics. Further investigation of these narratives is required in order to better understand why the developers of agricultural robotics may no longer perceive the technology as a “hot” ethical issue that should still be debated (Swierstra and Rip 2007), or address what Baur & Iles (2022) describe as “human vs human” conflicts and the frictions that may impact technological adoption.

## Conclusion

This study examined the nature of engagement in current agricultural robotics development processes in the UK, to investigate whether they are consistent with the aims of responsible innovation and just transitions encouraged by major policy and funding institutions. We offer insight into current approaches, identify the barriers met by those working in agricultural robotics development at present, and highlight some areas upon which future investigation could focus, such as limited inclusion and the non-ethical focus of development processes. The observations made in this study are specific to the UK national context for agricultural robotics development, and are based on a sample of a small population of developers working in the UK. However, the findings of this study reflect the prevailing narratives of research into agricultural technology and responsible innovation, particularly in considering issues of wider citizen inclusion and engagement.

On the ‘who’ of inclusion – this study finds that certain types of stakeholders are frequently engaged, including some growers/farmers, supply chain actors, policymakers, researchers, and insurers, but our analysis did not shed light on whether heterogeneity within groups was accounted for. There are a range of other stakeholders both from within and without the food industry that appear to be less engaged with, including agricultural workers, potentially ‘harder-to-reach' farmers, citizens, farm animals, and the living environment. Barriers to inclusion of a wider range of stakeholders are partially practical; for example, language barriers can make including migrant workers difficult, whilst more-than-human views are less immediately tangible, and the time needed to plan and deliver engagement opportunities can be prohibitive.

On the ‘how’ of inclusion – engagement that does happen tends to be through demonstrations, meetings, farm visits, and formal events, which are useful methods for working with some communities of policy and practice, but fail to include a wider set of deliberative methods that can harness different knowledge types.

On the ‘why’ of inclusion – the purpose of stakeholder engagement is focused on questions of technical sophistication and adoption, rather than explicitly designed to address ethical questions – although some ethical questions may be raised or addressed through engagement activities primarily focused on technical design (Rubambiza et al. 2022). Therefore, the explicit ethical motive inherent in responsible innovation is presently missing from UK agricultural robotics development practices.

In light of our findings, we make a number of suggestions for future research and delivery of stakeholder inclusion in the context of agricultural robotics. We suggest that a practical method for individual developers to undertake more substantive inclusion would be to undertake a stakeholder mapping exercise using a tool such as Mepham’s Ethical Matrix, such as we have done in this study (see Fig. 3). We recognise that all stakeholder groups cannot necessarily be included in all projects and for every question or problem, but such an exercise should invite reflexive questioning of whether planned engagement ensures that marginalised stakeholders and different types and scales of knowledge are included. We also recommend that developers collaborate with experts from the social sciences and humanities who are working on inclusive co-design, as seen in New Zealand in a collaborative project to develop robots for viticulture and horticulture (Legun and Burch 2021). Cross-disciplinary collaboration, training, and guidance from funders can also help to shift mindsets towards a wider appreciation of why stakeholders should be included substantively in development.

At a larger scale, policy-makers and funders can play a key role in setting the conditions through which trans-disciplinary collaborations can thrive, and in overcoming challenges reported in existing collaborations around agricultural technology (Alexander et al. 2023; Prutzer et al. 2023). We thus argue that our suggestions should be considered particularly by policy-makers, funders, and research institutions who set the structures within which agricultural innovation happens. In order to facilitate just transitions to sustainable agriculture, the structures of innovation (e.g. funding rules to encourage trans-disciplinarity, project assessment, incentive structures, knowledge support for innovators) should be adapted to make substantive inclusion easier to achieve and more attractive to pursue.

However, we recognise the possible naivety in the situation within and the position from which we make these structural recommendations. Critical social science on agri-tech ‘revolutions’ has brought to the fore how narratives around robotics and precision agriculture can express the ‘normative grammar’ of intensive, capitalist production (Miles 2019). Further, concerns regarding technocentric development (Eastwood et al. 2019), agri-food sanitisation (Reisman 2021), and digital solutionism (Guthman and Butler 2023) all highlight the role that narratives of technology development play in shaping approaches to innovation by individual decision makers. Although incentivising responsible innovation is a valuable contribution, it is important to account for the ways in which those in decision-making positions – be they technology developers, politicians, or investors – have the potential to use this greater power to realise radical change for just transitions, but equally to perpetuate the status quo as part of a normative narrative of technology development.

In conclusion, just transitions for sustainable agriculture are an explicit goal of major institutions from the United Nations to the European Commission. In order to facilitate these just transitions, multiple potential pathways for the use or non-use of technologies such as agricultural robotics must be explored, to avoid falling into a pattern of digital solutionism that fails to consider alternative approaches (Guthman and Butler 2023; McGrath et al. 2023). Responsible innovation of agricultural robotics can bring substantial benefits in opening up conversations to allow deeper reflection on if, and how, robotics can create a sustainable, more just food production system. If public and private developers of agricultural robotics are to pursue responsible innovation, as mandated by some of the public funding they receive from government, substantive inclusion of stakeholders is required.

## Data Availability

For reasons of confidentiality, the interview transcripts have not been made available.
